# Pathogenicity and tissue distribution of grass carp reovirus after intraperitoneal administration

**DOI:** 10.1186/1743-422X-11-178

**Published:** 2014-10-07

**Authors:** Hong-Ru Liang, Yong-Gang Li, Wei-Wei Zeng, Ying-Ying Wang, Qing Wang, Shu-Qin Wu

**Affiliations:** Pearl River Fisheries Research Institute, Chinese Academy of Fishery Sciences, Key Laboratory of Fishery Drug Development,Ministry of Agriculture, Key Laboratory of Aquatic Animal Immune Technology, Guangzhou, 510380 China; College of Fisheries and Life Science, Shanghai Ocean University, Shanghai, 201306 China

**Keywords:** Distribution, GCRV, qRT-PCR

## Abstract

Grass carp reovirus (GCRV) is the causative agent of grass carp hemorrhage and causes significant loss of fingerlings. However, little is known about how the virus is distributed in organs and tissues. The aim of the present study was to investigate the distribution of different GCRV stains in tissues and organs of grass carp. The pathogenicity and tissue distribution of GCRV were monitored after intraperitoneal administration. The study showed a distribution of GCRV in different tissues and organs, particularly in the liver, spleen, kidney, intestine, and muscle, which had a higher number of viral RNA copies during the sixth to ninth days. The kidney had the highest numbers of viral RNA copies, as high as 24000 copies. Until the fourteenth day, nearly no viral RNA copies could be detected. This study defined the virus distribution in different tissues of grass carp inoculated by i.p. and supplied clues for the pathogenesis of GCRV.

## Introduction

Grass carp reovirus (GCRV), a fatal pathogen to aquatic animals, can provoke severe hemorrhagic disease in grass carp and causes a mortality rate of up to 85% during the summer months, when water temperatures range between 24°C and 30°C [1, 2]. GCRV infects grass carp, *Ctenopharyngodon idellus*, was found to be capable of infecting black carp *Mylopharyngodon piceus*, topmouth gudeon, *Pseudorasbora parva*, and rare minnow *Gobiocypris rarus*, and historically has resulted in large losses in freshwater fish culture [3]. The disease is one of the major diseases that damages the grass carp breeding industry and, every year, leads to at least 1 billion economic losses in China.

GCRV belong to genus Aquareovirus in the family Reoviridae. The GCRV is a noneveloped, icosahedral particle comprised of 11 double-stranded RNA genome segments surrounded by multiple concentric protein capsids [4]. There is little know about the genus Aquareovirus, but always focused on the molecular and structural biology of their structural proteins [5].

However, to date, there is no effective antiviral treatment against GCRV infection [6], and preventing these infectious diseases is still a great challenge in grass carp farming [7]. GCRV has been recognized as the most pathogenic amongst in the isolated aquareoviruses [8]. However, information on the tissue distribution and dynamic changes of GCRV in tissues and organs after infection by GCRV is lacking. Moreover, GCRV serves as a good model for studying viral replication and pathogenesis of Aquareovirus due to its high virulence [9].

In this study, grass carp were intraperitoneally inoculated (i.p.) with different GCRV strains, and the tissue distribution was analyzed. The GCRV-HZ08 stain was isolated form China’s Zhejiang province and GCRV-CL stain was isolated form China’s Hunan province, which have different virulence. A better understanding of the viral dynamics and distribution of the different GCRV strains in tissues will be helpful to control the disease.

## Methods

### Cells, virus and reagents

GSB cells were used for GCRV replication and grown in Eagle’s minimum essential medium (MEM; Invitrogen, USA) supplemented with 10% fetal calf serum (FBS; HyClone, USA) at 37°C.

The GCRV-HZ08 and GCRV-CL strains were obtained from the Pearl River Fishery Research Institute (Guangzhou, China). Grass carp, Sixteen- to eighteen-week-old, with a body length of 10 ± 1.0 cm and an average weight of 20.0 ± 1.1 g, were obtained from a GCRV-free zone in NanHai (Guangdong, China).

### Immunofluorescence analysis

The cells were infected with GCRV-HZ08 or GCRV-CL at a multiplicity of infection (MOI) of 0.5. At 48 h post-infection, the infected cells were fixed with 4% paraformaldehyde for 20 min at room temperature and then treated with 0.1% Triton-X100. After washing 3 times with PBS, the cells were incubated with rabbit anti-grass carp reovirus antibody (1:1000), followed by FITC-conjugated goat anti- rabbit IgG (Boster, China) (1:100). The infected cells were determined using a fluorescence microscope.

### Fish pathogenicity experiments

Grass carps (ten in each group) were intraperitoneally (i.p.) injected with 0.2 mL of the appropriate GCRV-HZ08 or GCRV-CL, and the control group was intraperitoneally (IP) injected with 0.2 mL of PBS. The grass carps were monitored over three weeks for clinical signs of hemorrhagic disease.

### Tissue distribution of grass carp reovirus

The grass carps were divided into three groups, with 60 fishes in each group: (i) grass carps intraperitoneally inoculated with 100 μl GCRV-HZ08; (ii) grass carps intraperitoneally inoculated with 100 μl GCRV-CL; and (iii) grass carps intraperitoneally inoculated with sterile PBS as a negative control group.

### Detection of antibody

The blood of three grass carps from each group were sampled, and the titers of the serum were determined using Enzyme-linked immunosorbent assay (ELISA). For ELISA, 96-well plates were coated with 70 ng of the purified GCRV S10 protein per well at 4°C overnight. After all of the coated wells were blocked with 1% BSA, serum were loaded into the plate wells and incubated at 37°C for 2 h. Anti-grass carp monoclonal antibody with HRP conjugate (1:10000 dilution in 1% PBST) was then added and incubated at 37°C for 1 h. Finally, 3,3’,5,5’-tetramethylbenzidine (CoWin Biotech, Beijing) was used for the color reaction, which was stopped with H_2_SO_4_. Each serum was assayed in duplicate with three repeats. The absorbance was determined at 450 nm using a spectrophotometer (Infinite M200 PRO, Tecan).

### Detection of viral RNA copy numbers by real-time qRT-PCR

Three grass carps from each group were sampled from the first to fourteenth day, and then on the twenty-first and twenty-eighth day, and the different tissues were collected. All the samples were stored at -80°C until use.

The viral RNA copy numbers were measured by quantitative real-time RT-PCR (qRT-PCR) as in a previous study. Briefly, total RNA was extracted using the Trizol reagent (Invitrogen, NY, USA) according to the manufacturer’s protocol. The isolated RNA was then reverse transcribed using the First Strand cDNA Synthesis Kit (Takara, Japan). The cDNA was used as template for qRT-PCR performed using Premix Ex TaqTM (Perfect Real Time) (Takara, Japan) to assay samples with the following GCRV S7 gene-specific primers: F, 5’-ccaggaatcaatagcaatc-3’, and R, 5’-cctgatataatcgctcttc-3’. The internal probe was 5’-cgataaccaccactacggctg-3’. The probe was labeled on its 5’ end with FAM and on its 3’ end with Eclipse. To estimate virus replication, virus-specific mRNA expression was measured using qRT-PCR and expressed as the number of RNA copies per mg of tissue. The results were analyzed using the Applied Biosystems 7500 software.

### Statistical analysis

The experimental data were expressed as the mean ± SD. The data were analyzed by a one-way ANOVA analysis and Student's t-tests using the SPSS 13.0 statistical software.

## Results

### Immunofluorescence analysis

**Figure 1:**
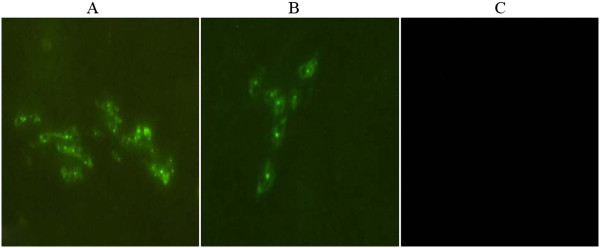
**Immunofluorescence analysis. A**: GSB cells were infected with GCRV- CL; **B**: GSB cells were infected with GCRV-HZ08; **C**: Uninfected GSB cells.

GSB cells were infected with GCRV-HZ08 or GCRV-CL and the number of infected cells was determined using a fluorescence microscope (Figure 1). Both of the GCRVstains could propagated in the GSB cells.

### Fish pathogenicity experiments

**Figure 2:**
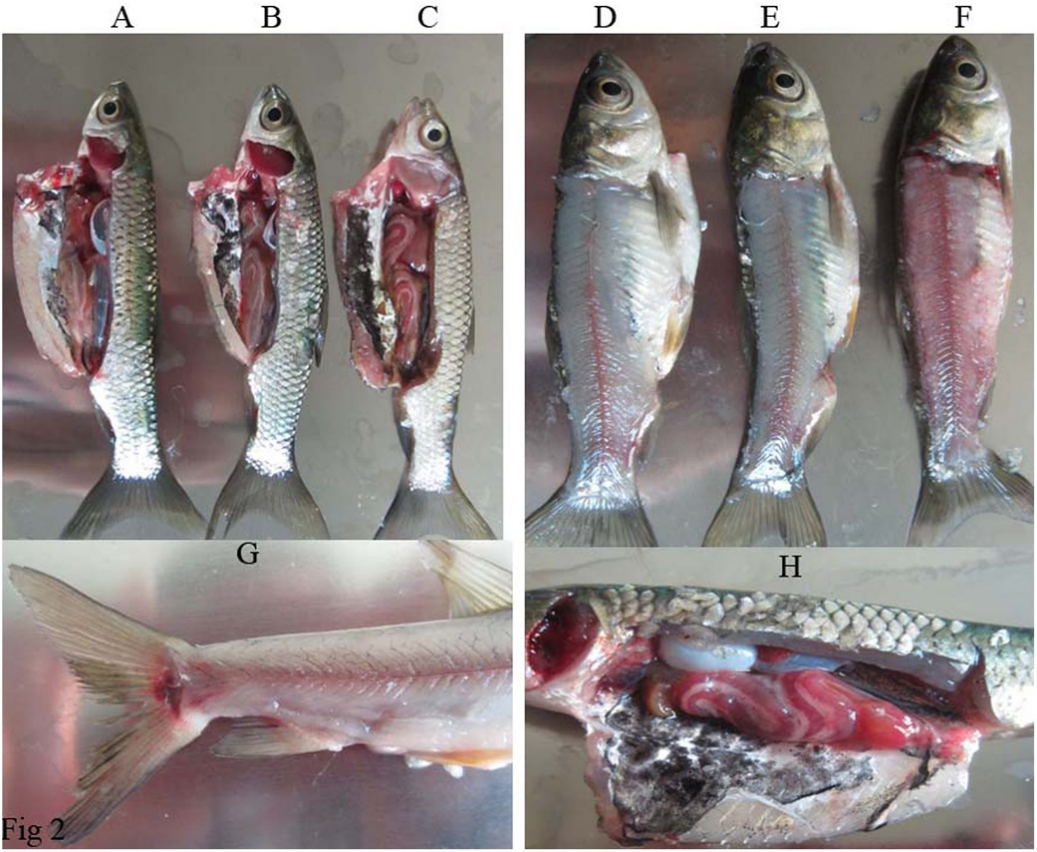
**Grass carps were infected by i.p. A/D**: Grass carps were injected with PBS; **B/E**: Grass carps were infected with GCRV-ZH08; **C/F**: Grass carps were infected with GCRV-CL; **G**: Grass carps infected with GCRV-CL showed hemorrhage at the base of fins; **H**: Grass carps infected with GCRV-CL showed hemorrhage in intestines.

Grass carps infected with GCRV-CL by i.p. showed spots or plate hemorrhages in the tissues or organs and fish died, such as hemorrhages at the base of fins, intestines, and muscle (Figure 2C/F/G/H). However, grass carps infected with GCRV-HZ08 (Figure 2B/E) or the PBS group (Figure 2A/D) did not show clinical signs of this disease. GCRV-CL strain was more virulent than the GCRV-HZ08 strain.

### Detection of antibody

**Figure 3:**
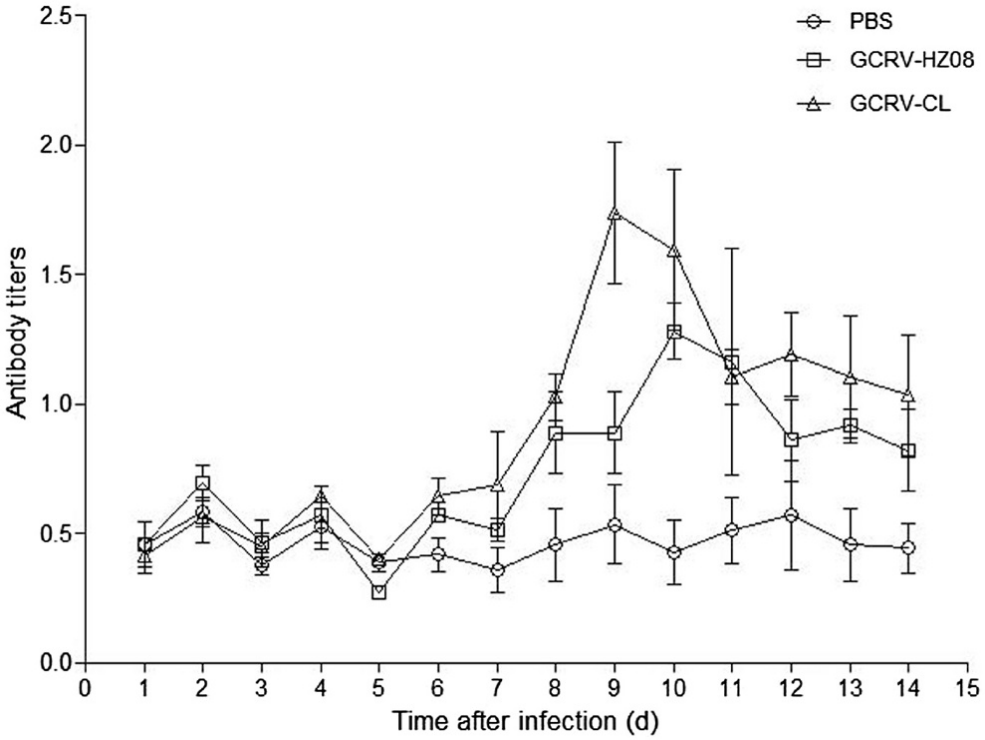
**Titers of the serum were determined by using ELISA.**

The blood of three grass carps from each group were sampled, and the titers of the serum were determined using ELISA (Figure 3). The groups infected by GCRV-HZ08 and GCRV-CL showed elevated antibody levels, which suggested that GCRV-HZ08 and GCRV-CL had infected the grass carp and induced the elevated antibody levels.

### Tissue distribution of grass carp reovirus

Three grass carps from each group were sampled from the first to fourteenth day and then on the twenty-first and twenty-eighth days. The viral RNA copy numbers of the samples in different tissues and at different times were detected by real-time qPCR.

### Liver

**Figure 4:**
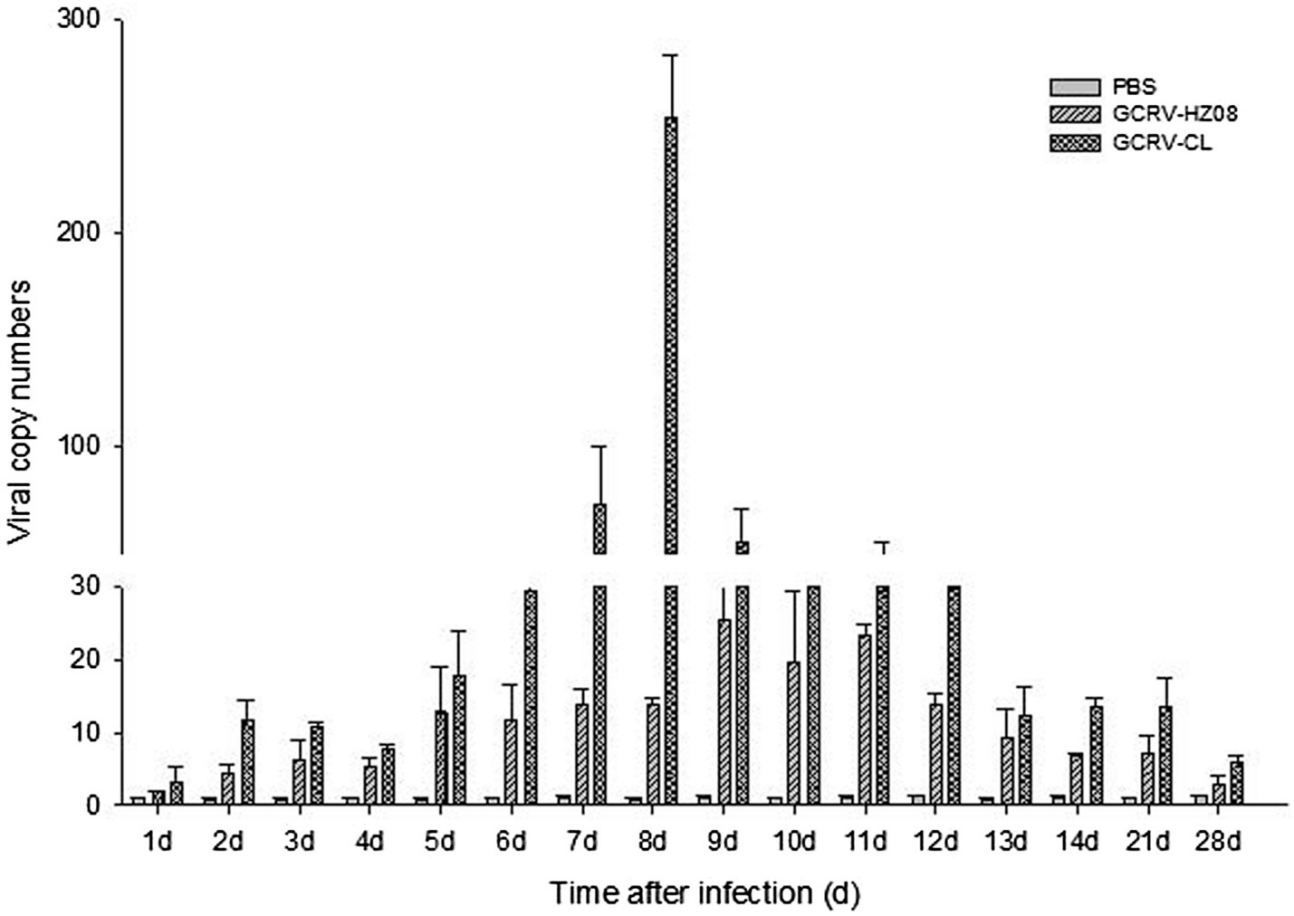
**The viral RNA copy numbers of the liver detected by real-time qPCR.**

To determine the viral RNA copy numbers in the liver, samples of liver were analyzed by real-time qPCR (Figure 4). The results showed that grass carps infected with GCRV-CL had the highest number of virus on the eighth day after infection, and the numbers on the seventh and ninth days were also high. No more than 30 viral copy numbers could be detected for GCRV-CL on the other days of infection or for GCRV-HZ08 on all days after infection.

### Spleen

**Figure 5:**
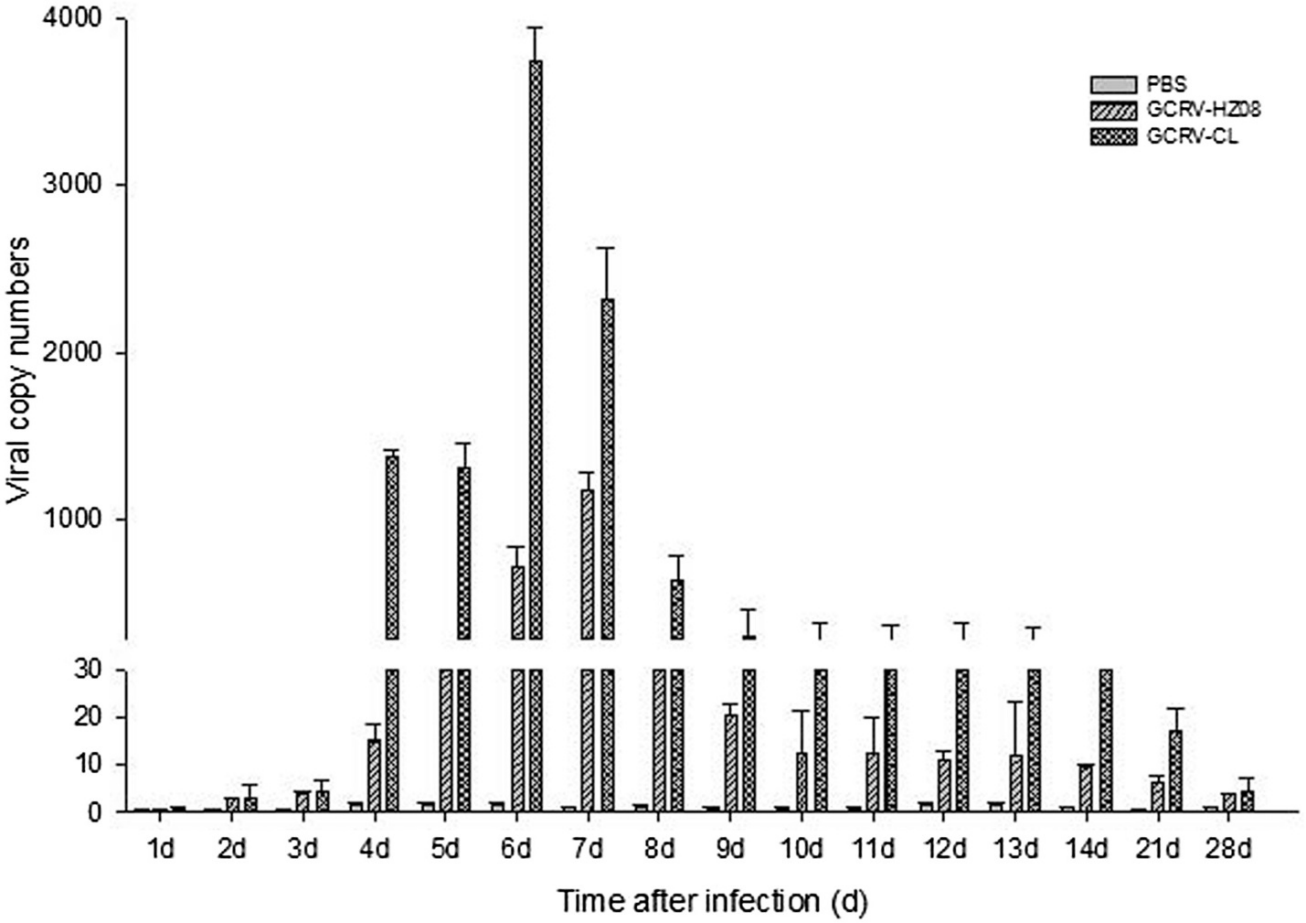
**The viral RNA copy numbers of the spleen detected by real-time qPCR.**

The viral RNA copy numbers of the spleen were detected using real-time qPCR (Figure 5). The results showed a higher number of GCRV-CL from the fourth to eighth days, whereas GCRV-HZ08 had a higher number of virus only on the sixth and seventh days, and the viral RNA copy numbers were less than that of GCRV-CL. Moreover, the highest number of virus was for GCRV-CL on the sixth day.

### Kidney

**Figure 6:**
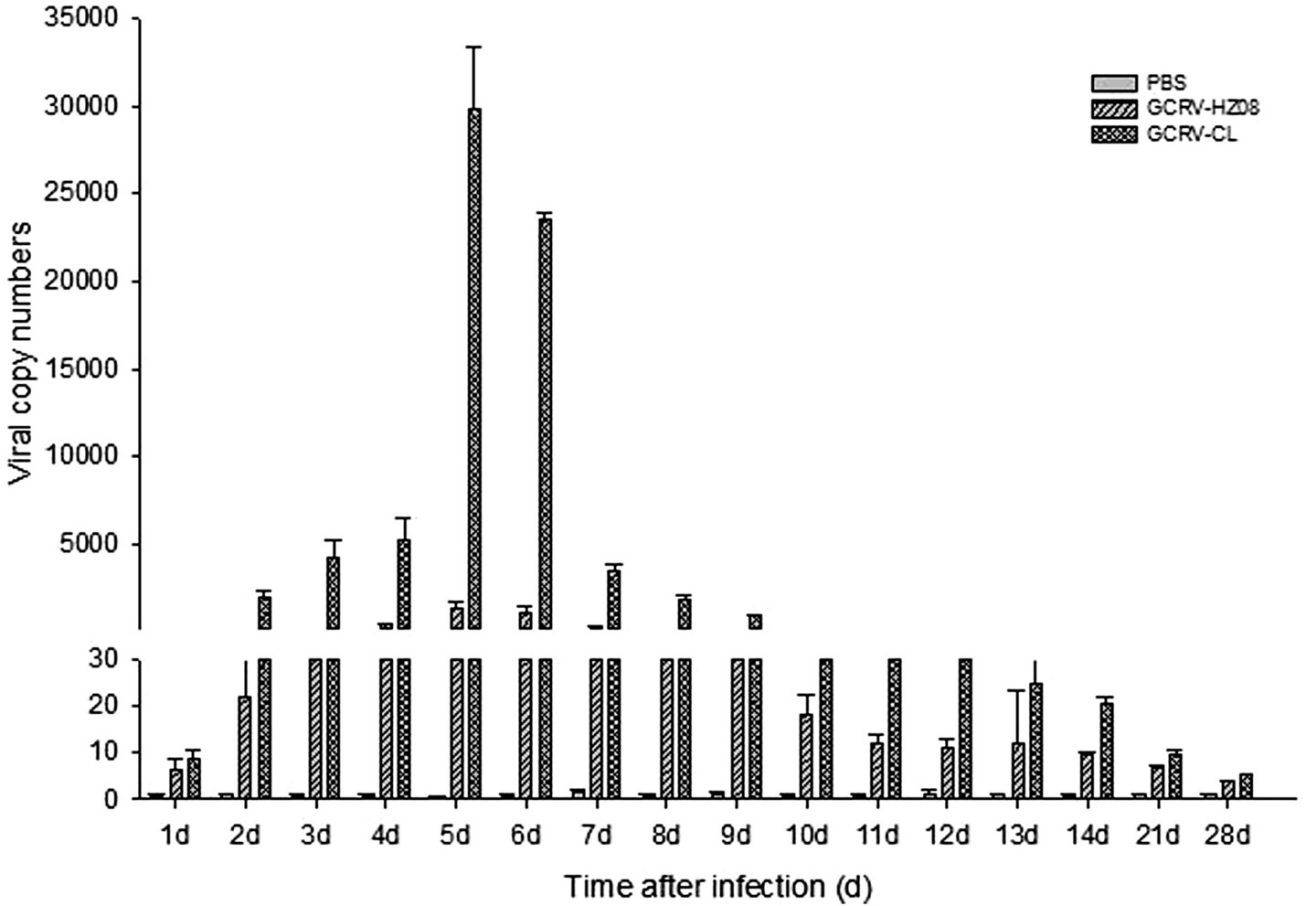
**The viral RNA copy numbers of the kidney detected by real-time qPCR.**

To determine the viral RNA copy numbers of the kidney, the samples were analyzed by real-time qPCR (Figure 6). The results showed that GCRV-CL had a higher viral number from the second to ninth day and the highest number on the fifth day. GCRV-HZ08 had a higher number of virus from the fourth to seventh day.

### Intestine

**Figure 7:**
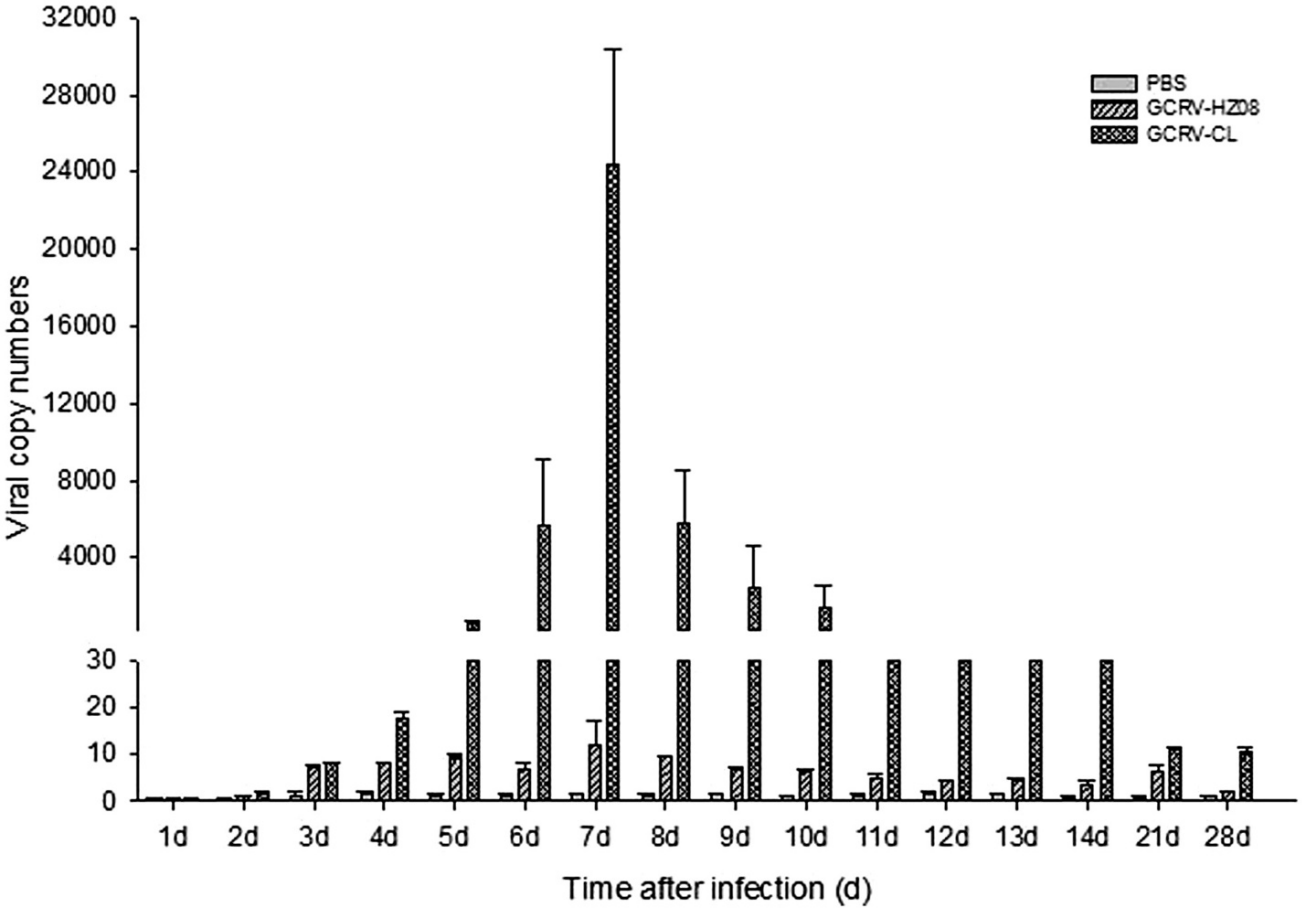
**The viral RNA copy numbers of the intestine detected by real-time qPCR.**

The viral RNA copy numbers of the intestine were determined by real-time qPCR (Figure 7). The results showed that GCRV-CL had a higher number of virus from the fifth to tenth day and the highest number of virus on the seventh day. GCRV-HZ08 did not have a higher number of virus on any day.

### Muscle

**Figure 8:**
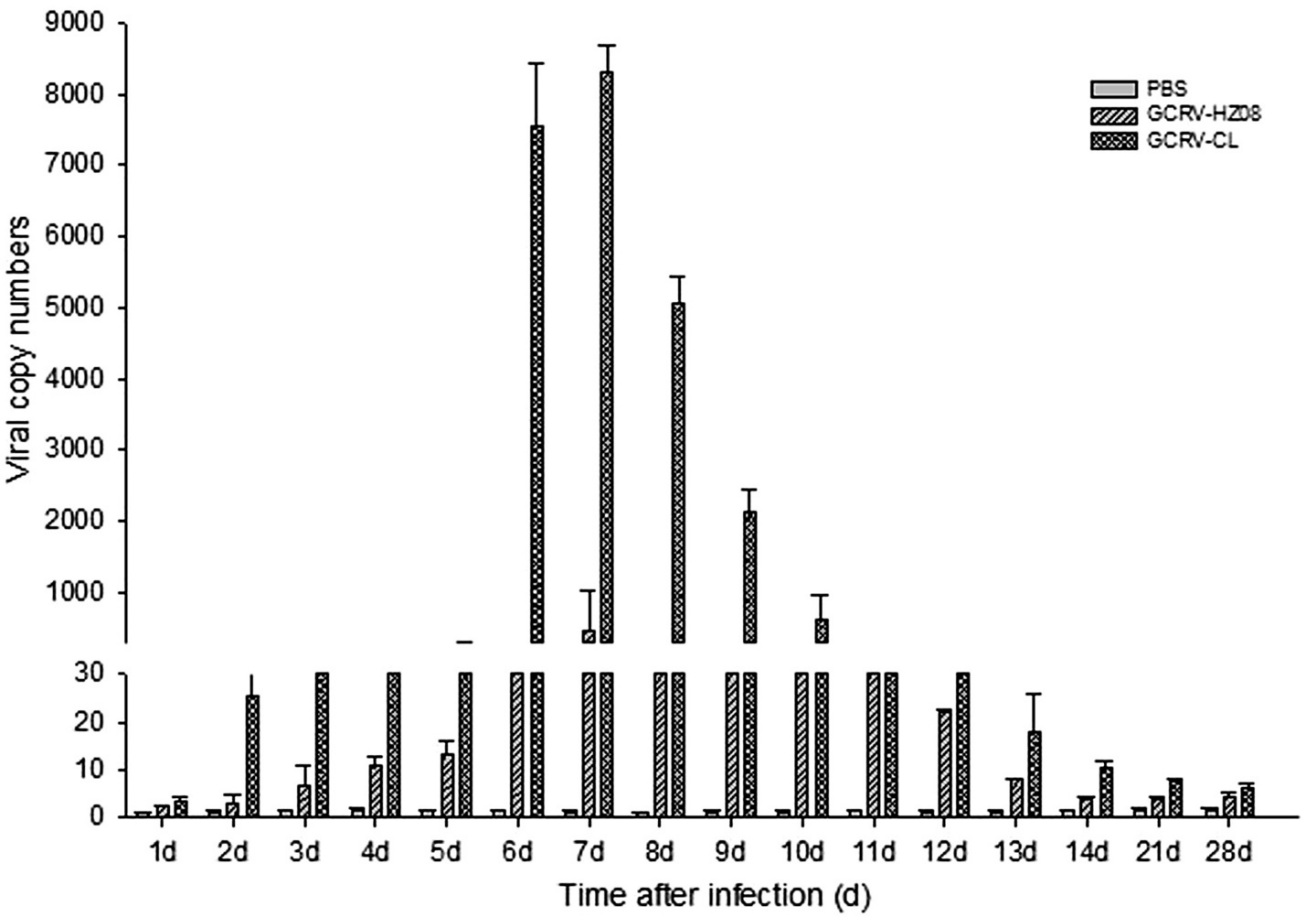
**The viral RNA copy numbers of the muscle detected by real-time qPCR.**

To determine the viral RNA copy numbers in the muscle, muscle samples were analyzed by real-time qPCR (Figure 8). The results showed that GCRV-CL had a higher number of virus from the sixth to tenth day and the highest number on the seventh day. GCRV-HZ08 had a higher number of virus in the seventh day.

## Discussion

GCRV is a multilayered, spherically structured particle that contains a genome of 11 segments of dsRNA (named S1–S11) that encode 7 structural (VP1–VP7) and 5 nonstructural proteins [10]. But it is still limited to know the diseases [3].

Most attenuated virus strains replicate quickly and express large amounts of antigen protein, thereby inducing strong adaptive immune responses that result in rapid virus clearance. But pathogenic virus strains replicate at a lower rate than attenuated strains and they could reach their target tissues [11, 12]. In this study, GCRV-CL was a pathogenic strain, and GCRV-HZ08 was an attenuated virus strain. Hence, GCRV-HZ08 may be cleared from the body more easily, which could explain why GCRV-HZ08 was undetectable or only detected for a few days by qRT-PCR.

The clinical signs of this infection are hemorrhages of organs, showing spots or plate forms, in combination with some or all of the following signs: exophthalmia, body darkening, hemorrhage of the mouth cavity, hemorrhagic or pale gills, and hemorrhage at the base of fins and gill covers. Internal hemorrhages may occur throughout the musculature, liver, spleen, kidney, and intestines. However, these are only some of the signs; and sick fingerlings show one or all of them [13]. The results of this study were consistent with the above-mentioned study. Grass carps infected with GCRV-CL by i.p. showed spots or plate hemorrhages in the tissues or organs, such as hemorrhage at the base of fins, intestines, and muscle, but grass carps infected with GCRV-HZ08 did not show any of the clinical signs (Figure 2). GCRV-HZ08 could have been attenuated after cell passaging; therefore, the GCRV-CL strain was more virulent than the GCRV-HZ08 strain. Western blotting showed elevated antibody levels and suggested that GCRV-CL and GCRV-HZ08 had infected the grass carps and induced the high antibody titers (Figure 3).

In this study, the viral RNA copy numbers were detected by qRT-PCR, and threshold cycles (Ct) for each sample that were lower than 35 (Ct < 35; copy > 20) were treated as positive. Grass carp infected with GCRV-CL showed higher numbers of viral RNA copies during the sixth to ninth day in nearly all tissues, indicated that the seventh day was the peak incidence. These four days had at least more than 1000 copies. The kidney had the earliest highest numbers of viral RNA copies, which were as high as 24,000 copies on the fifth day. The kidney may be a region of virus replication, serving as a factory to produce a large number of GCRV that are then spread to other parts of the body. Ten days after infection, the viral RNA copy numbers gradually decreased, and until the fourteenth day, nearly no viral RNA copies could be detected, possibly because GCRV-CL was gradually cleared from the body. The virus may have been eliminated in the fourteenth day.

In conclusion, this study defined the virus distribution of different tissues of grass carp inoculated by i.p. and supplies clues for the pathogenesis of GCRV. However, further studies should be performed to better understand the tropism and transmission of GCRV.

## Conclusions

Two different strain of GCRV:GCRV-CL and GCRV-HZ08 were infected grass carp. Grass carps infected byGCRV-CL showed spots or plate hemorrhages in the tissues and was more virulent than the GCRV-HZ08 strain. The grass carps infected by GCRV-CL had higher viral RNA copy numbers than infected by GCRV-HZ08 in different tissues and organs, particularly in the liver, spleen, kidney, intestine, and muscle, which had a higher number of viral RNA copies during the sixth to ninth days. But ten days after infected, there were not high viral RNA copy numbers. This study defined the virus distribution of different tissues of grass carp inoculated by i.p. and supplies clues for the pathogenesis of GCRV.
